# HIV-Infected Children in Rural Zambia Achieve Good Immunologic and Virologic Outcomes Two Years after Initiating Antiretroviral Therapy

**DOI:** 10.1371/journal.pone.0019006

**Published:** 2011-04-28

**Authors:** Janneke H. van Dijk, Catherine G. Sutcliffe, Bornface Munsanje, Pamela Sinywimaanzi, Francis Hamangaba, Philip E. Thuma, William J. Moss

**Affiliations:** 1 Macha Research Trust, Macha Hospital, Choma, Zambia; 2 Department of Epidemiology, Bloomberg School of Public Health, Johns Hopkins University, Baltimore, Maryland, United States of America; University of Cape Town, South Africa

## Abstract

**Background:**

Many HIV-infected children in sub-Saharan Africa reside in rural areas, yet most research on treatment outcomes has been conducted in urban centers. Rural clinics and residents may face unique barriers to care and treatment.

**Methods:**

A prospective cohort study of HIV-infected children was conducted between September 2007 and September 2010 at the rural HIV clinic in Macha, Zambia. HIV-infected children younger than 16 years of age at study enrollment who received antiretroviral therapy (ART) during the study were eligible. Treatment outcomes during the first two years of ART, including mortality, immunologic status, and virologic suppression, were assessed and risk factors for mortality and virologic suppression were evaluated.

**Results:**

A total of 69 children entered the study receiving ART and 198 initiated ART after study enrollment. The cumulative probabilities of death among children starting ART after study enrollment were 9.0% and 14.4% at 6 and 24 months after ART initiation. Younger age, higher viral load, lower CD4+ T-cell percentage and lower weight-for-age z-scores at ART initiation were associated with higher risk of mortality. The mean CD4^+^ T-cell percentage increased from 16.3% at treatment initiation to 29.3% and 35.0% at 6 and 24 months. The proportion of children with undetectable viral load increased to 88.5% and 77.8% at 6 and 24 months. Children with longer travel times (≥5 hours) and those taking nevirapine at ART initiation, as well as children who were non-adherent, were less likely to achieve virologic suppression after 6 months of ART.

**Conclusions:**

HIV-infected children receiving treatment in a rural clinic experienced sustained immunologic and virologic improvements. Children with longer travel times were less likely to achieve virologic suppression, supporting the need for decentralized models of ART delivery.

## Introduction

More than 90% of the 2 million children living with HIV worldwide reside in sub-Saharan Africa [1]. With increasing evidence that pediatric treatment programs in resource-poor settings can achieve treatment outcomes comparable to those of developed countries [2], [3], most countries in sub-Saharan Africa have implemented policies to increase access to antiretroviral therapy (ART). However, considerable inequities in access to treatment remain [4], as most HIV services are concentrated in urban areas distant from where many HIV-infected children reside.

Rural clinics and residents face unique barriers to care and treatment. Shortages of health care personnel, equipment and drugs may more heavily affect rural clinics, and several studies identified limited modes of transportation and food security as factors affecting the ability of rural residents to access care and treatment [5], [6], [7], [8]. These factors may impact treatment responses of children. A recent study comparing HIV-infected children receiving ART in urban and rural Zambia found higher levels of undernutrition and mortality throughout treatment in the rural clinics [9]. To minimize barriers to care, different models of service delivery have been implemented in rural areas to increase accessibility of ART, including the use of nurses [10], [11], [12], [13], [14], [15] and general practitioners [16] in the provision of ART at primary care clinics, and home-based care provided by trained field officers [17] and volunteers [18]. Consequently, evaluation of treatment programs and treatment outcomes in rural settings is needed. Several reports are available from rural programs [10], [12], [13], [14], [15], [16], [17], [18], [19], but few focus on children [5], [11].

This observational cohort study reports immunologic and virologic treatment outcomes and mortality among HIV-infected children receiving up to two years of treatment at a rural HIV clinic in Macha, Zambia.

## Materials and Methods

### Ethics Statement

The study was approved by the Ministry of Health in Zambia, the Research Ethics Committee of the University of Zambia and the Institutional Review Board of the Johns Hopkins Bloomberg School of Public Health. Written informed consent was obtained from parents or guardians and assent was obtained from children 8–16 years of age.

### Study setting and population

The study was conducted at Macha Hospital in rural Southern Province, Zambia. The study setting has been described in detail elsewhere [5]. Briefly, the catchment area of Macha Hospital is populated primarily by traditional villagers living in small, scattered homesteads. Macha Hospital is a 208-bed district-level referral hospital administered by the Zambian Brethren in Christ Church that functions within the healthcare system of the Ministry of Health. Since 2005, Macha Hospital has been one of the primary ART providers in the district and has cared for over 6000 HIV-infected adults and children through the Government of Zambia's antiretroviral treatment program, with additional support from the President's Emergency Plan for AIDS Relief (PEPFAR) through the non-governmental organization AidsRelief.

HIV-infected children are referred to the clinic from voluntary counseling and testing programs, in- and outpatient clinics, and rural health centers. Since February 2008, children born to HIV-infected women are routinely tested for HIV infection at approximately 6 weeks of age using dried blood spot samples transported to Lusaka, Zambia for HIV DNA PCR. Clinical care is provided without charge by medical doctors and clinical officers, and adherence counseling by nurses and trained counselors. Eligibility for ART is determined based on WHO treatment guidelines [20], [21]. The first-line antiretroviral treatment regimen consists of two nucleoside reverse transcriptase inhibitors (lamivudine plus zidovudine, stavudine or abacavir) and a non-nucleoside reverse transcriptase inhibitor (efavirenz or nevirapine). Fixed dose pediatric combinations of lamivudine and stavudine are available, as well as Triomune® baby and junior.

### Study procedures

HIV-infected children younger than 16 years of age seeking care at the antiretroviral treatment clinic at Macha Hospital were eligible for enrollment into a prospective, observational cohort study beginning in September 2007. Children were evaluated at study visits approximately every three months, at which time a questionnaire was administered to the guardian, the child was examined and a blood specimen was obtained. The questionnaire included information on demographic information, household characteristics, medical history, HIV-related stigma and adherence for children receiving treatment. Adherence was measured at each visit by pill count for children receiving pills and by weight for children receiving syrups. Blood specimens were collected in EDTA tubes. As part of routine clinical care, CD4^+^ T cell counts and percentages were measured using the Guava Easy CD4 system (Guava Technologies, Inc., Hayward, CA). As part of study procedures, plasma levels of HIV RNA were quantified by a reverse transcriptase polymerase chain reaction assay (Amplicor HIV-1 Monitor v. 1.5, Roche Molecular Systems; lower limit of detection: 400 copies/mL). Viral load testing was only performed for children who started ART after study enrollment and was performed on batched samples; therefore, results were not available for clinical care. Information prior to study enrollment was abstracted from medical records. Home visits were attempted for children who missed study visits. For children who died, the location and cause of death were ascertained through verbal autopsy or through hospital or clinic records.

### Statistical analysis

For the present analysis, all children enrolled in the study and receiving ART between September 2007 and September 2010 were included. This study sample consisted of two groups of children: 1) children receiving ART at study enrollment (Group A); and 2) children who were treatment-naïve at study enrollment and initiated ART during the specified period (Group B). Children were included in the analysis until they died, were lost to follow-up or were administratively censored at September 1, 2010 or 24 months of ART. Children whose last study visit occurred more than 6 months prior to September 1, 2010 were considered lost to follow-up.

Data were entered in duplicate using EpiInfo (Centers for Disease Control and Prevention) and analyses were conducted in SAS for Windows version 9.1 (SAS Institute Inc., Cary, NC) and STATA, version 9 (StataCorp LP, College Station, Texas). Characteristics at study enrollment and ART initiation were compared between groups of children. The Wilcoxon rank sum test was used to compare continuous variables and the chi-square test was used to compare categorical variables. A measure of socio-economic status (SES) was calculated based on the Demographic and Health Survey SES scale used in Zambia [22]. SES percentiles were based on the predetermined cutoffs (<25^th^ = 0–6; 26–50^th^ = 7–12; 51–75^th^ = 13–18; >75^th^ = 19–24). Weight-for-age z-scores were calculated based on the WHO growth standards [23] and children with z-scores below −2 were defined as underweight. Severe immunodeficiency was defined by age according to the 2006 WHO treatment guidelines [20], and severe anemia was defined as hemoglobin concentration <8 g/dL [24]. If laboratory tests were not available from the visit at which ART was initiated, results were used within 3 months prior to the date of initiation.

Risk factors for mortality after ART initiation were evaluated using Cox proportional hazards models. Time since ART initiation was used as the time axis, and late entries were used for children in Group A. Risk factors at ART initiation of interest included age, sex, orphan status, travel time to the clinic, CD4+ T-cell percentage, WAZ, anemia and viral load. Factors associated with mortality (p<0.10) in the univariable models were eligible for inclusion in the multivariable model.

Immunologic and virologic treatment outcomes as well as adherence were assessed. For all outcomes, children were included if they had at least one post-ART measure available. To report outcomes at specific time points, measurements were aggregated to within 45 days. Immunologic treatment outcomes were evaluated using linear mixed effects models with random intercept, exchangeable correlation structure and robust standard error estimation. A spline term was added at 7.5 months, the upper window around the 6-month measure. Adherence was calculated for each drug returned and is reported as the percentage of medication returned of the expected use since the prior visit. For children taking more than one drug, the minimum adherence of all drugs was calculated. Adherence measures were capped at 100%. Both continuous and categorical measures of adherence are reported. For the categorical measure, a child was defined as adherent if they took more than 95% of the prescribed doses.

Predictors of viral suppression between 6 and 24 months after ART initiation were assessed among children in group B. As children could contribute more than one measure of viral load, repeated measures logistic regression models were fit with generalized estimating equations. Predictors of interest included age, sex, orphan status, education of the primary caregiver, socio-economic status, travel time, ART regimen, underweight and severe immunodeficiency at ART initiation. Adherence and the presence of others in the household receiving ART were also assessed at the time of each viral load measure. As the sample size was small, separate multivariable models were built for each predictor found to be associated with viral suppression (p<0.10) in the univariable models. Only factors associated with the predictor of interest were included in each multivariable model.

## Results

### Characteristics of the study population at study enrollment and ART initiation

Between September 2007 and September 2010, 267 children received ART, including 69 children who entered the study already receiving ART (Group A) and 198 children who initiated ART after study enrollment (Group B). Children in Group A entered the study a median of 8.0 months (IQR: 2.3, 12.9) after initiating ART, while children in Group B initiated ART a median of 1.7 months (IQR: 0.9, 5.0) after study enrollment. The median follow-up time in the study was 14.9 months (IQR: 5.0, 19.2) for children in Group A and 11.8 months (IQR: 4.4, 20.7) for children in Group B. The median age of children at enrollment into the ART clinic was 2.76 years (interquartile range [IQR]: 1.42, 7.67) among children in Group A and 2.03 years (IQR: 1.11, 4.95) among children in Group B (p = 0.07). The median age of children at study enrollment was 3.80 years (IQR: 2.26, 8.54) among children in Group A and 2.21 (IQR: 1.27, 5.36) in Group B, and 49.1% were male (Table 1). At ART initiation, the median age was 2.90 years (IQR: 1.71, 7.75) in Group A and 2.69 (IQR: 1.50, 5.92) in Group B. The proportion of children who were underweight at ART initiation was 66.7% and 53.4% among children in Group A and B, respectively. The proportion of children with severe immunodeficiency was 73.7% and 60.9% among children in Group A and B, respectively.

**Table 1:** Characteristics at study enrollment and ART initiation of HIV-infected children receiving antiretroviral therapy.

	Total (n = 267)	Group A (n = 69)	Group B (n = 198)	p-value
***Study enrollment***				
Median age in years (IQR)	2.67 (1.41, 6.38)	3.80 (2.26, 8.54)	2.21 (1.27, 5.26)	0.001
<2 yrs	104 (39.0)	14 (20.3)	90 (45.5)	
2–4.9 yrs	86 (32.2)	29 (42.0)	57 (28.8)	
≥5 yrs	77 (28.8)	26 (37.7)	51 (25.8)	0.001
Male sex (%)	131 (49.1)	41 (59.4)	90 (45.5)	0.05
Mother received PMTCT (%)	14 (5.3)	1 (1.5)	13 (6.6)	0.19
Vital status of parents (%)				
Both alive	189 (72.1)	41 (62.1)	148 (75.5)	
One parent died	52 (19.9)	15 (22.7)	37 (18.9)	
Both died	21 (8.0)	10 (15.2)	11 (5.6)	0.03
Travel time (hours)(%)				
<1	24 (9.1)	6 (9.1)	18 (9.1)	
1–2	80 (30.4)	21 (31.8)	59 (30.0)	
3–4	88 (33.5)	19 (28.8)	69 (35.0)	
≥5	71 (27.0)	20 (30.3)	51 (25.9)	0.80
***ART initiation***				
Median age in years (IQR)	2.73 (1.57, 6.73)	2.90 (1.71, 7.75)	2.69 (1.50, 5.92)	0.37
<1 yr	39 (14.6)	10 (14.5)	29 (14.7)	
1–1.9 yrs	59 (22.1)	12 (17.4)	47 (23.7)	
2–4.9 yrs	87 (32.6)	23 (33.3)	64 (32.3)	
≥5 yrs	82 (30.7)	24 (34.8)	58 (29.3)	0.69
Median WAZ (IQR)^b^	−2.28 (−3.37, −1.39)	−2.48 (−3.64, −1.72)	−2.16 (−3.22, −1.34)	0.10
Underweight (%)	120 (56.6)	34 (66.7)	86 (53.4)	0.10
Missing (%)	55 (20.6)	18 (26.1)	37 (18.7)	
Median CD4% (IQR)	15.9 (10.5, 20.1)	13.1 (9.4, 16.2)	16.8 (10.9, 20.6)	0.03
Severe immunodeficiency (%)^b^	140 (63.1)	28 (73.7)	112 (60.9)	0.14
Missing (%)	45 (16.9)	31 (44.9)	14 (7.1)	

Group A: children who entered the study already receiving ART; Group B: children who initiated ART after study enrollment.

a Among respondents who were primary caregivers (n = 247).

b Among children <10 years of age.

c Defined by age according to the 2006 WHO guidelines.

Few mothers reported receiving antiretroviral drugs to prevent mother-to-child transmission (5.3%). Children were primarily cared for by a parent (78.4%), although 27.9% reported that at least one parent had died. Other primary caregivers included grandparents (11.7%) and aunts or uncles (7.2%). The education level of the primary caregiver was low, with 5.7% reporting no education and 58.5% reporting no more than a primary school education, 52.1% of whom completed grade 7. Only 35% reported a secondary school education. In addition, 80.9% of caregivers reported being able to read either Tonga or English. The majority (65.8%) of children lived in households with a SES in the lowest quartile and 30% lived in households with a SES in the second lowest quartile. Children who initiated ART prior to study enrollment were more likely to be male, to have lost both parents, and to have initiated ART with a lower median CD4^+^ T-cell percentage (Table 1).

### Mortality

Within the first two years after ART initiation, 27 children (10.1%) died (Group A: 5 (7.3%); Group B: 22 (11.1%); p = 0.36), 13 children (4.9%) transferred to another clinic (Group A: 2 (2.9%); Group B: 11 (5.6%); p = 0.38), and 4 children (1.5%) defaulted (Group A: 1 (1.5%); Group B: 3 (1.5%); p = 0.97). The proportion of children who defaulted among those enrolled for more than 6 months prior to September 1, 2010 was 1.7%.

The majority of deaths occurred within the first few months of starting ART. Among children initiating ART after study enrollment (Group B), the median time to death from ART initiation was 3.08 months (IQR: 0.59, 6.33). Among all children, the cumulative probabilities of death were 9.0% (95% CI: 5.8, 13.9), 12.0% (95% CI: 8.1, 17.5) and 14.4% (95% CI: 9.9, 20.6) at 6, 12, and 24 months after ART initiation, respectively. Information on cause of death was available for 18 children. Contributing factors included diarrhea (n = 9), malnutrition (n = 8), tuberculosis (n = 5), pneumonia (n = 5), meningitis (n = 2), cerebral malaria (n = 1), encephalopathy (n = 1), and possible Kaposi's sarcoma (n = 1). Location of death was available for 21 children. Ten children died in the hospital (47.6%), two children died on the way to the hospital (9.5%), one child died in the rural health center (4.8%), and eight children died at home (38.1%). Younger age, lower WAZ, lower CD4+ T-cell percentage and high viral load (>750,000 copies/mL) at ART initiation were associated with a higher risk of mortality (Table 2). Only younger age and lower WAZ remained significantly associated with higher mortality after adjusting for other factors.

**Table 2:** Risk factors for mortality after initiation of antiretroviral treatment.

Characteristics at ART initiation	Crude hazard ratio (95% CI)	Adjusted hazard ratio (95% CI)
Female	1.03 (0.48, 2.20)	
Age	0.79 (0.66, 0.95)	0.40 (0.22, 0.74)
<1 yr	8.72 (1.79, 42.47)	
1–1.9 yrs	9.81 (2.21, 43.66)	
2–4.9 yrs	2.44 (0.47, 12.58)	
≥5 yrs	1	
Orphan	0.68 (0.26, 1.79)	
Travel time		
<1 hr	1	
1–2 hrs	2.91 (0.37, 22.96)	
3–4 hrs	2.22 (0.28, 17.57)	
≥5 hrs	2.93 (0.37, 23.48)	
WAZ^a^	0.55 (0.43, 0.72)	0.64 (0.48, 0.86)
≥−2	1	
−2.1 to −3	3.68 (0.67, 20.13)	
<−3	10.96 (2.49, 48.25)	
Hemoglobin <8 g/dL	1.97 (0.66, 5.91)	
CD4 percentage (per 5 points)	0.77 (0.56, 1.05)	0.67 (0.44, 1.02)
Severe immunodeficiency^b^	1.23 (0.49, 3.08)	
Viral load >750,000 copies/mL^c^	19.35 (2.31, 161.90)	3.03 (0.32, 28.58)

a Among children <10 years of age.

b Defined by age according to the 2006 WHO guidelines.

c An indicator for missing viral load was included in the multivariable model as viral load at ART initiation was only available on a subset of children.

### Immunologic and virologic treatment outcomes

**Figure 1:**
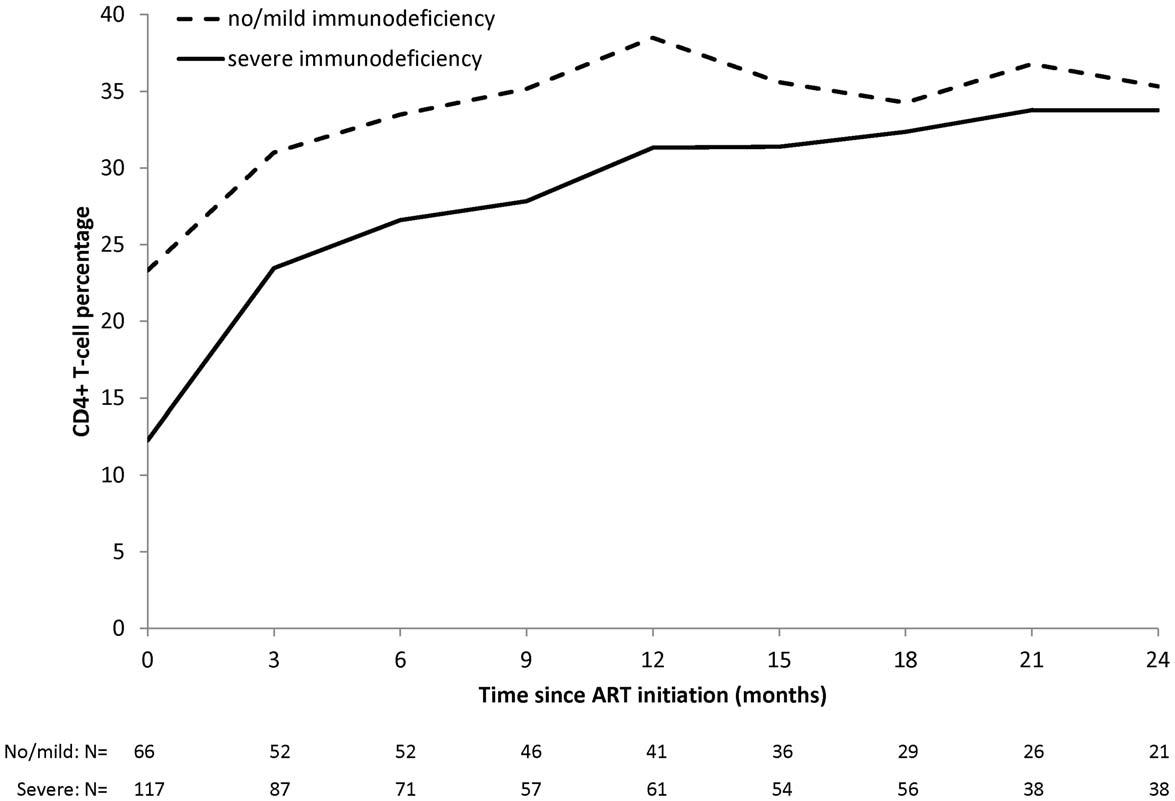
Mean CD4+ T-cell percentage (95% CI) after ART initiation, by level of immunodeficiency at initiation.

Among children with at least one post-ART measure, immunologic and virologic treatment outcomes improved within three to six months of starting ART (Table 3). Mean CD4^+^ T-cell percentage increased from 16.3% at treatment initiation to 29.3%, 33.9%, 33.0% and 35.0% at 6, 12, 18 and 24 months on ART. Consequently, the proportion of children with a CD4^+^ T-cell percentage >25% increased from 10.9% at treatment initiation to 66.7%, 81.5%, 84.5% and 87.5% at 6, 12, 18, and 24 months on ART. Results of the longitudinal models indicated that CD4^+^ T-cell percentage increased by 1.80 (standard error [SE]: 0.088; p-value = <0.0001) percentage points per month in the first 6 months, and then increased by 0.23 (SE: 0.058; p-value = <0.0001) percentage points per month thereafter. No difference in the pattern of improvement was found by age, sex or underweight status at ART initiation. Monthly increases in CD4^+^ T-cell percentages in children with severe immunodeficiency at ART initiation were significantly greater than in children without severe immunodeficiency, both within the first 6 months (2.04 [SE: 0.11], p-value = <0.0001 vs. 1.60 per month [SE: 0.15], p-value = <0.0001; p-interaction = 0.02) and after 6 months of ART (0.30 [SE: 0.09], p-value = 0.001 vs. 0.075 [SE: 0.07], p-value = 0.28; p-interaction = 0.04) (Figure 1).

**Table 3:** Immunologic and virologic outcomes and adherence by time on treatment.

	Treatment initiation	3 months	6 months	12 months	18 months	24 months
***Immunologic outcome***	*N = 183*	*N = 149*	*N = 135*	*N = 119*	*N = 97*	*N = 72*
Mean CD4% (STD)	16.3 (7.6)	26.6 (11.0)	29.3 (10.4)	33.9 (9.6)	33.0 (9.0)	35.0 (8.5)
Mean change in CD4% from ART initiation (STD)	—	9.4 (8.9)	12.4 (8.5)	17.4 (8.8)	17.3 (9.9)	19.0 (9.2)
% with CD4%>25%	10.9	54.4	66.7	81.5	84.5	87.5
% missing, of those in care^a^	7.8	27.7	25.0	23.7	20.5	20.0
***Virologic outcome*** b	*N = 106*	*N = 100*	*N = 96*	*N = 77*	*N = 53*	*N = 27*
Median log VL (IQR)	5.4 (5.0, 5.9)	2.6 (2.6, 2.6)	2.6 (2.6, 2.6)	2.6 (2.6, 2.6)	2.6 (2.6, 2.6)	2.6 (2.6, 2.6)
Median change in log VL from ART initiation (IQR)	—	−2.7 (−3.3, −2.2)	−2.7 (−3.3, −2.1)	−2.6 (−3.3, −1.8)	−2.6 (−2.8, −1.8)	−2.4 (−2.7, −1.1)
% with undetectable VL	—	90.0	88.5	88.3	86.8	77.8
% missing, of those in care^a^	46.5	45.1	35.1	29.4	30.3	34.1
***Adherence***		*N = 150*	*N = 133*	*N = 115*	*N = 87*	*N = 71*
Median (IQR)	—	98.5 (89, 100)	98 (93, 100)	99 (96, 100)	100 (96, 100)	99 (95, 100)
% ≤95%	—	35.3	32.3	23.5	24.1	25.4
% missing, of those in care^a^		27.2	26.1	26.3	28.7	21.1

Note: Immunologic and virologic treatment outcomes were evaluated among children with at least one post-ART measure available.

a Children in care were defined as those who were enrolled in the study and presented to the clinic at the specified visit or, if the visit was missed, at any subsequent visit.

b The analysis was restricted to children in group B for the virologic outcome.

Median viral load decreased rapidly after the start of ART, from 5.4 log copies/mL at treatment initiation to <2.6 log copies/mL throughout treatment (Table 2). The proportion of children with undetectable viral load increased to 90.0%, 88.5%, 88.3%, 86.8% and 77.8% at 3, 6, 12, 18 and 24 months on ART. One hundred and four children received ART for at least 6 months, 90 (87%) of whom had persistent undetectable viral loads at or beyond 6 months of ART. Fourteen children (13%) had at least one sample with detectable viral load, of whom 4 (29%) had persistent detectable viral load, 3 (21%) had detectable viral load at more than one visit, 6 (43%) had viral rebound which then remained undetectable, and 1 (7%) had a detectable viral load on their last available sample.

The 4 children with persistent detectable viral load had no evidence of clinical or immunologic failure, and no children in group B were switched to a second line regimen due to treatment failure during the study period. One child in group A was switched to a second line regimen including a protease inhibitor due to clinical and immunologic failure 20.7months after initiation.

### Adherence

Adherence data were available for at least one study visit for 216 children (Group A: 55; Group B: 161). Estimated adherence, defined as the minimum adherence of all drugs taken, over the study period was high, with a median of 98–100% at all time points up to 24 months after treatment initiation (Table 2). The proportion of children who were non-adherent (adherence percentage less than 95%) decreased over time, from 35.3% at 3 months to 25.4% at 24 months. We examined patterns of adherence over time to determine if the same children were consistently non-adherent. Among the 172 children who had at least 2 adherence measures at study visits, 74 (43%) were consistently adherent, 3 (2%) were consistently non-adherent, and 95 (55%) had patterns alternating between adherence and non-adherence. Adherence was not associated with any characteristics of the child or caregivers. Children taking ART regimens including fixed dose combinations were, however, less likely to be non-adherent (e.g. 45.2% of children taking individual drugs were non-adherent compared to 7.1% of children taking fixed dose combinations 9 months after ART initiation; p = 0.01). Non-adherence was also marginally associated with a lower risk of achieving viral suppression (odds ratio: 0.68; 95% CI: 0.46, 1.01; p = 0.05).

### Predictors of viral suppression after 6 months of ART

**Figure 2:**
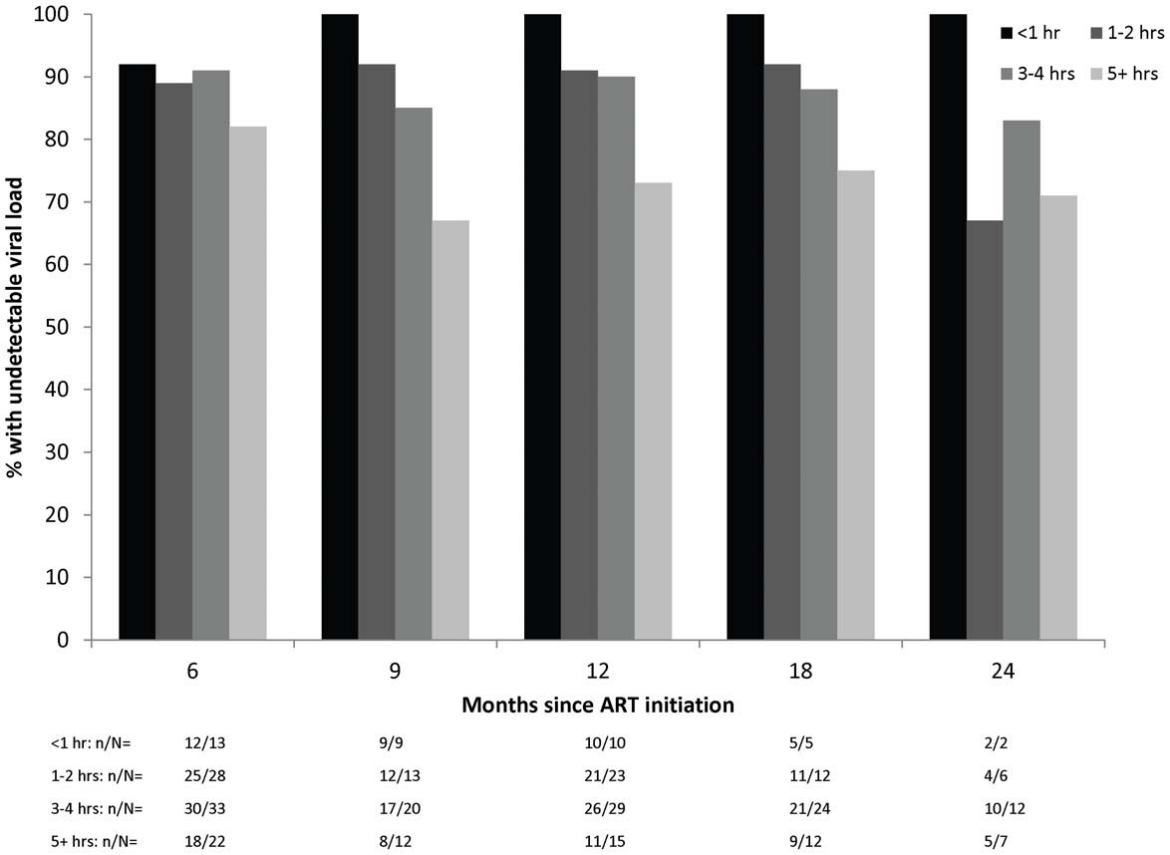
Percentage of children achieving viral suppression after 6 months of ART, by travel time.

Longer travel time, younger age, non-orphan status and use of nevirapine at ART initiation were significantly associated with a lower risk of viral suppression (Table 4; Figure 2). These characteristics were correlated, as children with shorter travel times were more likely to be older and to be orphans. Children receiving nevirapine were also more likely to be younger. After adjustment, longer travel times and use of nevirapine remained marginally associated with a lower risk of viral suppression.

**Table 4:** Predictors of viral suppression after 6 months of antiretroviral therapy.

	Crude odds ratio (95% CI)	Adjusted odds ratio (95% CI)
**Characteristics at ART initiation**		
Female	0.69 (0.25, 1.94)	
Age		
<1 yr	0.32 (0.05, 2.07)	1.01 (0.05, 2.82)
1–1.9 yrs	0.16 (0.03, 0.84)	0.85 (0.14, 4.95)
2–4.9 yrs	0.39 (0.07, 1.98)	1.08 (0.10, 11.35)^d^
≥5 yrs	1	1
Orphan	7.22 (1.61, 32.45)	3.32 (0.64, 17.30)^e^
Travel time		
<1 hr	1	1
1–2 hrs	0.34 (0.04, 3.16)	0.51 (0.07, 3.86)
3–4 hrs	0.20 (0.03, 1.65)	0.36 (0.05, 2.37)
≥5 hrs	0.10 (0.01, 0.82)	0.17 (0.02, 1.19)^e^
High SES (>50^th^ percentile)	2.28 (0.35, 15.00)	
Education of the primary caregiver		
None/Primary school	1	
Secondary school/university	1.22 (0.40, 3.78)	
Underweight^a^	0.87 (0.28, 2.68)	
Severe immunodeficiency^b^	0.70 (0.24, 2.03)	
High viral load (>750,000 copies/mL)^c^	0.60 (0.17, 2.04)	
ART regimen including stavudine	0.47 (0.15, 1.48)	
ART regimen including nevirapine	0.35 (0.46, 0.98)	0.33 (0.11, 1.03)^f^
ART regimen including fixed dose combinations	2.30 (0.65, 8.10)	
**Characteristics at each viral load measure**		
Other people in the household on ART	0.78 (0.48, 1.27)	

a Among children <10 years of age.

b Defined by age according to the 2006 WHO guidelines.

c An indicator for missing viral load was included in the multivariable model as viral load at ART initiation was only available on a subset of children.

d Multivariable model included orphan status, travel time, nevirapine and age at ART initiation.

e Multivariable model included orphan status, travel time and age at ART initiation.

f Multivariable model included age and nevirapine at ART initiation.

## Discussion

This study of treatment outcomes among HIV-infected children in Zambia demonstrated that ART could effectively be delivered in this rural setting and that these children responded well to treatment. Two groups of children were assessed, those who entered the study receiving ART and those who initiated ART during the study period. Children in the latter group were younger and had better clinical and immunological profiles. While the children receiving ART at study entry represented a subset of children who initiated ART at the clinic prior to the start of this study, these findings are consistent with a previous study in this population demonstrating improvements in the enrollment and treatment of children at younger ages and less severe stages of disease progression [25].

The proportion of children who died (14.4%) within 24 months of starting ART was higher than reported from other studies of children in sub-Saharan Africa. The KIDS-ART-LINC study in 17 sites across sub-Saharan Africa reported cumulative probabilities of mortality of 4.8%, 6.0% and 6.0% at 6, 12 and 24 months [26], respectively, while the International epidemiologic Databases to Evaluate AIDS in Southern Africa (IeDEA-SA) study across 10 sites in southern Africa reported probabilities of 4.5% and 7.7% at 12 and 36 months [27], [28], respectively. This difference may partially be explained by the relatively younger and more undernourished rural population in this study, as both of these factors are associated with higher mortality [2]. However, the difference may also be explained by the lower observed loss to follow-up of 1.5% at 24 months in this study, compared to 10.3% in the KIDS-ART-LINC study over the same time period [26]. Among adults, approximately 40% of losses to follow-up represented unreported deaths [29]. In fact, when the IeDEA-SA study accounted for mortality among losses to follow-up, the 12 month mortality increased from 4.5% to 8.7% [28]. Consequently, the high mortality and low loss to follow-up in this study could also be due to more complete ascertainment of outcomes as a result of contact tracing. The majority of deaths occurred within the first 3 months of ART and were associated with younger age, lower WAZ, lower CD4^+^ T-cell percentage and higher viral load, consistent with other observations [2].

Children followed in this study achieved good immunologic and virologic treatment outcomes, such that 88% had a CD4^+^ T-cell percentage greater than 25% and 78% had an undetectable HIV viral load 24 months after initiating ART. Contrary to the results of an earlier cross-sectional analysis in this cohort [5], early gains in CD4^+^ T-cell percentages were maintained throughout follow-up. Similar improvements in immune status were observed in other African studies [2]. The level of viral suppression in this cohort, however, was higher than reported in many other studies of African children [2], [27]. Few studies are available for comparison from rural areas, but one study in rural South Africa reported that 71% of children remaining on treatment after 12 months achieved viral suppression [30].

Correlates of viral suppression were explored, including established factors such as drug regimen and adherence, and factors related to treatment in a rural setting, such as travel time to the clinic. Clinical trials in adults [31], [32] and observational studies in children [33], [34], [35] showed higher rates of virologic failure with nevirapine-based regimens compared to efavirenz-based regimens, consistent with our findings. Adherence in this cohort was high throughout treatment and was associated with viral suppression. However, despite being one of the main determinants of viral suppression after ART initiation, adherence has not consistently been found to correlate with viral suppression in children [36], [37], [38], perhaps due to the difficulties in measuring adherence among children, including complex drug regimens, use of pills and syrups, and the potential involvement of multiple caregivers in the administering of medication [39]. This study attempted to overcome these difficulties by using a more objective measure of adherence by medication returns rather than self-report.

The main novel factor associated with viral suppression was travel time. As this was a rural population, participants travelled from surrounding villages to the clinic and over a quarter of the study population reported travelling five or more hours. Transportation and distance have been reported as barriers to care and treatment in this [5] and other adult and pediatric populations [6], [7], [8], [40], as long travel distances or times incur a direct cost for transportation and indirect cost for time away from work and childcare. Distance to the clinic was associated with retention in this clinic, with children living farther away from the clinic more likely to be lost to follow-up [9]. However, this is the first report of an association between travel time and virologic outcome, with children travelling five or more hours significantly less likely to achieve viral suppression. This relationship is hypothesized to be due to the impact of travel time on the frequency of visits to the clinic and pharmacy. In this study, associations between travel time and missed or delayed visits as well as adherence were not found; however, information on pharmacy or clinic appointments in between study visits was not available. This finding should be confirmed in other populations as it has important implications for the treatment of HIV-infected children as treatment programs are increasingly implemented and scaled up in rural areas.

The main limitations of the study include the small sample size, particularly at longer follow-up times, and missing data on adherence and treatment outcomes, which may have led to an overestimation of the success of ART. However, this is one of the few pediatric studies conducted in rural sub-Saharan Africa, and the high proportion of children with available immunologic and virologic measures suggests minimal bias for these outcomes. In addition, children cared for at the Macha ART clinic benefit from being enrolled in a research study and attending a faith-based health facility, which may have higher levels of support and staffing than government clinics in the same region. While the study relied on laboratory tests (with the exception of viral load) and clinical measures performed as part of routine care to minimize this bias, the good treatment outcomes observed in this study may not be generalizable to other rural government clinics.

In summary, this study demonstrated that ART can effectively be delivered in a rural setting and that children experienced sustainable immunologic and virologic improvements after initiating ART. The observed detrimental effect of long travel times on virologic suppression may have implications for ART delivery in this setting. As treatment programs expand further into rural areas, clinic catchment areas will expand. Unless decentralized models of care are implemented, children may have to travel great distances for their care and treatment, which may impact both their ability to remain in care and respond to treatment.
